# The α-ketoglutarate dehydrogenase complex in cancer metabolic plasticity

**DOI:** 10.1186/s40170-017-0165-0

**Published:** 2017-02-02

**Authors:** Renaud Vatrinet, Giulia Leone, Monica De Luise, Giulia Girolimetti, Michele Vidone, Giuseppe Gasparre, Anna Maria Porcelli

**Affiliations:** 1 Dipartimento Farmacia e Biotecnologie (FABIT), Università di Bologna, Via Selmi 3, 40126 Bologna, Italy; 2 Dipartimento Scienze Mediche e Chirurgiche (DIMEC), U.O. Genetica Medica, Pol. Universitario S. Orsola-Malpighi, Università di Bologna, Via Massarenti 9, 40138 Bologna, Italy

**Keywords:** α-Ketoglutarate dehydrogenase complex, α-Ketoglutarate, Mitochondrial function, Metabolic stresses, Cancer plasticity, Cell signaling, Oncometabolite, Epigenetics

## Abstract

Deregulated metabolism is a well-established hallmark of cancer. At the hub of various metabolic pathways deeply integrated within mitochondrial functions, the α-ketoglutarate dehydrogenase complex represents a major modulator of electron transport chain activity and tricarboxylic acid cycle (TCA) flux, and is a pivotal enzyme in the metabolic reprogramming following a cancer cell’s change in bioenergetic requirements. By contributing to the control of α-ketoglutarate levels, dynamics, and oxidation state, the α-ketoglutarate dehydrogenase is also essential in modulating the epigenetic landscape of cancer cells. In this review, we will discuss the manifold roles that this TCA enzyme and its substrate play in cancer.

## Background

Cancer cells must acquire several biological properties in order to survive, proliferate, and disseminate. These functional features, or “hallmarks of cancer”, comprise sustaining proliferative signaling, evading growth suppressors, escaping cell death, and activating invasion/metastasis, all of which conspire to lead to pathologically high levels of cell survival and growth, and ultimately tumorigenesis [1]. In the last 10 years, an increasing number of studies suggests that cancer cells reprogram their metabolism in order to most effectively support growth and proliferation. Thus, metabolic rewiring has become an additional hallmark of cancer [1]. Increased aerobic glycolysis, as initially described by Otto Warburg [2], is observed in the majority of neoplasms in vivo, where it is believed to confer advantages to cancer cells for the production of energy, biomass, and reducing equivalents [3]. Although Warburg hypothesized that such a biochemical phenotype arises from the accumulation of mitochondrial defects, it is now known that, upon nutrient deprivation, oxidative metabolism can be promptly re-established, and a significant level of OXPHOS is maintained both in vitro and in vivo [4–9]. A common feature of solid tumors is that cells rapidly accumulate in bulks, with limited blood supply, and will hence cope with fluctuations in oxygen and nutrients, which will inevitably force them to modulate mitochondrial function consequently. Interestingly, it has been shown that neither hypoxia nor OXPHOS defects imply a complete shutoff of mitochondrial metabolism, and in either case the tricarboxylic acid (TCA) cycle may adjust metabolic fluxes to promote a glutamine-dependent biosynthetic pathway that sustains tumor progression [10]. Hence, the TCA cycle represents a metabolic hub that drives substrate utilization upon changes in resources availability. With respect to this, the discovery of mutations in genes encoding key enzymes of the TCA cycle has brought into light the importance of intracellular TCA cycle metabolite levels in modifying both the metabolic and the epigenetic landscape of cancer cells. Modification of TCA metabolic fluxes and metabolites levels in response to environmental pressures might therefore account for tumor adaptation and plasticity in the changing environment. In this frame, the α-ketoglutarate dehydrogenase complex (α-KGDC) stands out as being deeply interconnected with the respiratory chain, tightly regulated upon tumor microenvironmental changes, a modulator of the level of the signaling metabolite α-ketoglutarate (α-KG), a regulator of cellular redox state, and at the crossroads of numerous metabolic routes. In this review, we will discuss the manifold roles this enzymatic complex and its substrate α-KG play in cancer.

## Main text

### The α-KGDC in cell metabolism

The TCA cycle is fueled by substrates entering at different gateways to convey the carbon source for both energy production and biosynthesis. In the canonical view, acetyl CoA is provided by the oxidation of carbohydrates, mostly glucose and fatty acids, and is then condensed with oxaloacetate to form citrate. The subsequent series of oxidative reactions leads to the production of the reducing equivalents NADH and FADH_2_ that feed respiratory complex I (CI) and respiratory complex II (CII), respectively, to generate the mitochondrial membrane potential (Δψ_m_) required for ATP production. Glutamine, the most abundant amino acid in the plasma, has been widely described as an additional key source of both carbon and nitrogen, especially for fast proliferating cells [11]. Glutaminolysis results in the production of α-KG, either following dehydrogenation of glutamate or through a transamination reaction. In turn, α-KG can fuel both energetic and anabolic pathways: it may be oxidized by the α-KGDC inside the mitochondria or it may be reduced, thereby pushing the TCA cycle towards citrate [10, 12–14]. The latter may be extruded to the cytosol, where it may be converted back into acetyl CoA, and thereby used for lipid biosynthesis.

**Fig. 1:**
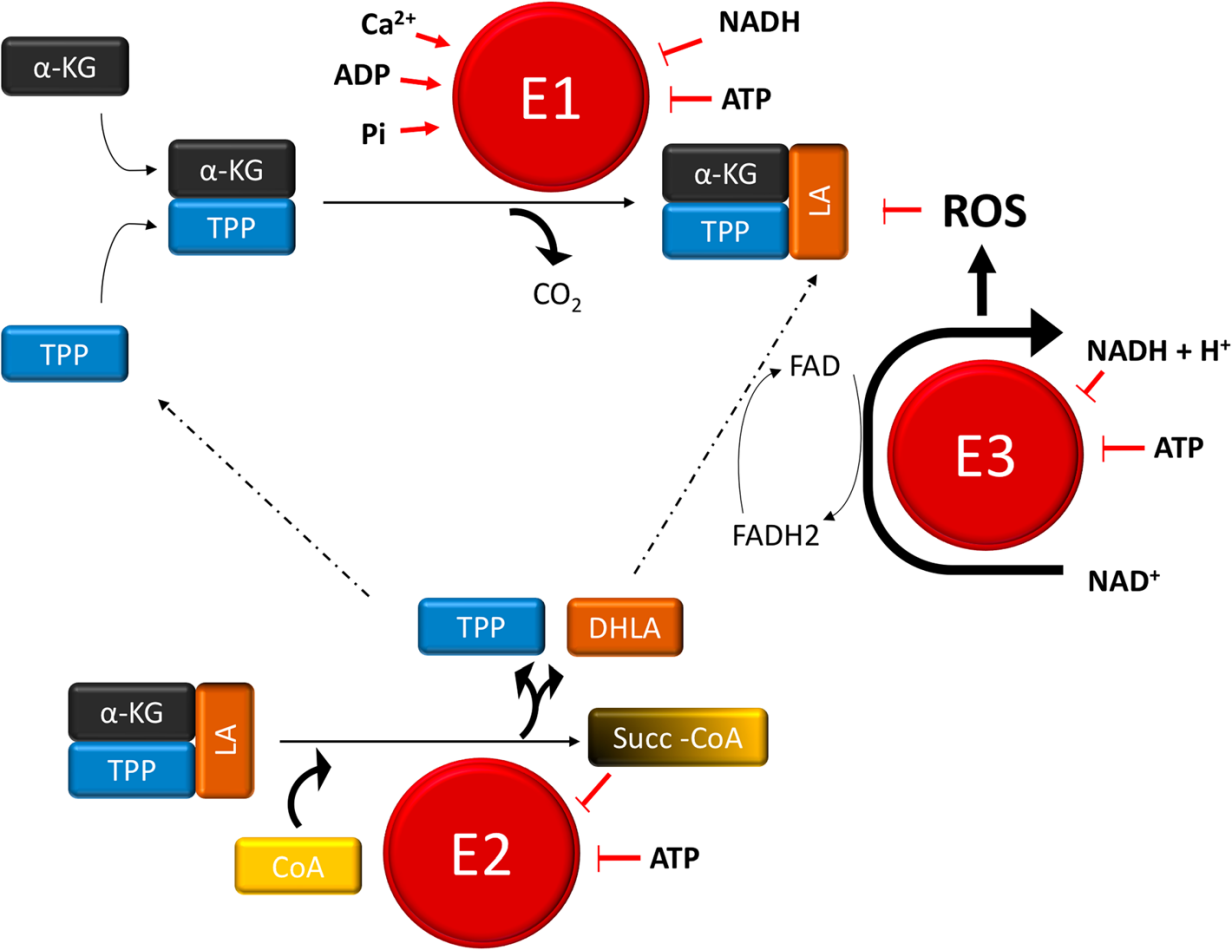
Regulation mechanisms of α-KGDC. α-KGDC is a complex consisting of multiple copies of three enzymes: α-﻿ketoglutarate dehydrogenase (E1), dihydrolipoamide succinyltransferase (E2), and dihydrolipoamide dehydrogenase (E3). α-KG reacts with the TPP that binds E1, and is thereby a decarboxylated-generating hydroxyethyl compound. E1 also catalyzes the transfer of two electrons and the acetyl group of the TPP on lipoic acid (LA), which is bound to the E2 subunit. The reaction performed by E2 is a transesterification in which the thiol group of the Coenzyme A (CoA) replaces the thiol group of E2, producing Succ-CoA and the reduced form of the lipoic group, the dihydrolipoic acid (DHLA). The subunit E3 catalyzes the transfer of two atoms of hydrogens from DHLA to its prosthetic group FAD, restoring oxidized LA. The reduced FADH_2_ of the E3 enzyme transfer H^+^ to NAD^+^ to form NADH.

Hence, the manifold mitochondrial functions allow cells to rely on different sources of nutrients for energetic and anabolic purposes. How cells balance the utilization of these nutrients depends on key metabolic enzymes, whose activities are modulated in response to genetics and environmental pressures. In this light, the α-KGDC lies at the very hub of metabolic pathways and its activity is finely regulated by the levels of ATP, ADP, inorganic phosphate (Pi), and by the NADH/NAD^+^ ratio, which are tightly dependent on the respiratory chain activity and on the rate of the glycolytic flux. The product of the α-KGDC reaction, succinyl CoA (Succ﻿-CoA), has been shown to exert a direct control on the enzyme activity along with calcium (Ca^2+^) and reactive oxygen species (ROS) (Fig. 1). Further, variations of mitochondrial pH and oxygen levels also appear to be involved in the regulation of the enzyme. Hence, the multifactorial modulation of α-KGDC function may reflect its key role in orchestrating the responses to the ever-changing metabolic requirements of a cancer cell.

### How the α-KGDC contributes to adapt mitochondrial metabolism to bioenergetic requirements

#### Structure and energetic regulation of the α-KGDC

In the TCA cycle, the α-KGDC catalyzes the reaction between α-KG and CoA, using thiamine pyrophosphate (TPP) as a cofactor and reducing the pyridine nucleotide NAD^+^ to NADH, finally generating succ-CoA and CO_2_. The α-KGDC is a multienzyme complex composed of three subunits (Fig. 1). The E1 subunit, encoded by the human *OGDH* gene, is a dehydrogenase that catalyzes the decarboxylation of α-KG, the first step required to produce succ-CoA. The second step is the reductive succinylation of the dihydrolipoyl groups, a reaction carried out by the E2 subunit, i.e., the dihydrolipoamide succinyltransferase, encoded by the human *DLST* gene*.* The E3 subunit, encoded by the human *DLD* gene, is the dihydrolipoamide dehydrogenase, which catalyzes the reoxidation of the E2 dihydrolipoyl groups, eventually reducing the final acceptor NAD^+^ to NADH [15]. The regulation of the α-KGDC highlights a dynamic interplay between the enzyme and the OXPHOS to adjust mitochondrial metabolism through cell energy status sensing. Both the E1 and the E3 subunits are inhibited by NADH [16], which accumulates following a decrease of CI function [17]. Indeed, the latter complex is the first and the largest of the respiratory chain and catalyzes NADH oxidation to transfer electrons to flavin mononucleotide, which are used to reduce coenzyme Q to ubiquinol (QH_2_). The latter is subsequently used by complex III to reduce cytochrome *c* in the mitochondrial intermembrane space (IMS), and complex IV uses cytochrome *c* to reduce molecular oxygen, which is the final electron acceptor [18]. Hence, CI actively participates to the generation of the electrochemical gradient by feeding the ETC to generate ATP, which makes NADH an essential substrate for oxidative metabolism. Interestingly, evidence is given for the existence of a direct interaction between CI and α-KGDC, which not only would provide an effective NADH oxidation mechanism via substrate channeling compared to free diffusion [19–21] but also implicates a higher sensitivity of α-KGDC to NADH levels, placing the enzyme on the front line to adapt to variations in ETC efficiency. In addition, a high ADP/ATP ratio and a high concentration of Pi independently enhance the activity of the α-KGDC, with a low ADP/ATP ratio having opposite effects [22, 23]. The levels of Pi and ADP are indicators of a low energetic condition, and both molecules act as positive effectors by increasing the affinity of the enzyme for its substrate. Conversely, higher ATP levels increase the amount of substrate necessary to reach the half-maximum rate of the enzyme, therefore reducing its activity [22, 24]. The regulation of α-KGDC by both the adenine nucleotide phosphorylation state and the NADH/NAD^+^ ratio is tightly dependent on the Δψ_m_: on the one side, ATP extrusion from the mitochondria to the cytosol is controlled by the ADP/ATP carrier that is regulated by high Δψ_m_ and exchanges ATP with ADP in a 1:1 ratio [25]. On the other side, in cases when the ETC is damaged, the production of mitochondrial NADH, driven by cytosolic reductive power, is decreased [26]. This implies that the energetic control on the α-KGDC might be exclusively mitochondrial, and that a feedback loop relying on both substrate and energy availability is triggered between the OXPHOS and the enzyme, thereby ensuring an optimal cooperation. In this light, it may be envisioned that a decrease in mitochondrial respiration, or a significant ATP accumulation, may be associated with a decrease in α-KGDC activity. Changes in the enzyme function would in turn balance mitochondrial NADH levels, thus modulating CI activity and thereby ATP production. However, it has been observed in human neuroblastoma cells that decreasing α-KGDC activity up to half its maximum decreases neither Δψ_m_ nor mitochondrial ATP levels [27]. In line with this, the existence of a threshold for the α-KGDC capacity has been demonstrated, which can be greatly inhibited before affecting the maximal mitochondrial oxygen consumption rate [28]. NADH levels can therefore vary broadly before becoming a limiting factor for cellular respiration, suggesting that any reduction of α-KGDC activity might represent a first attempt to adapt metabolism by modulating TCA flux, before impinging on ETC function.

#### Calcium-mediated regulation of the α-KGDC

The relationship between α-KGDC and Ca^2+^ further emphasizes the pivotal role of the enzyme in regulating cell metabolism. The mitochondria have long been thought to be a Ca^2+^ sink, with the main scope of regulating this cation homeostasis in cells. Cytosolic Ca^2+^ has been shown to foster NADH oxidation by the glycerol dehydrogenase to ultimately produce and import FADH_2_ within the mitochondria as a substrate for CII [29]. Furthermore, Ca^2+^ stimulates NADH production through the reactions of the TCA enzymes pyruvate dehydrogenase (PDH), isocitrate dehydrogenase (﻿IDH) and α-KGDC [16, 30]. Among these three enzymes, α-KGDC has been shown to be the most responsive to Ca^2+^, as the cation lowers the enzyme *K*_M_ for α-KG [22, 25, 29, 31]. Interestingly, the sensibility of α-KGDC to Ca^2+^ depends on the concentration of NADH, and on the ATP/ADP ratio, again highlighting a regulatory role of the ETC on the enzyme activity. Indeed, an increase in both cofactors lowers α-KGDC stimulation by Ca^2+^ [24]. Also, calcium enters the mitochondria through a uniporter, a process driven by the negative potential across the IMM [32]. With respect to this, cancer cells have been shown to be particularly sensitive to an arrest of mitochondrial metabolism through the inhibition of Ca^2+^ transfer into the mitochondria. Indeed, while normal cells slow down proliferation when Ca^2+^ import is inhibited, cancer cells proceed through mitosis and end up necrotic, a route that may be rescued through dimethyl-α-KG supplementation [33]. This finding indicates that cancer cells ought to rely on a functional TCA cycle to sustain successful proliferation, and that α-KG may help overcome calcium shortage and sustain a minimal OXPHOS activity, or may drive adaptive responses to oxidative metabolism impairment.

#### pH-mediated regulation of the α-KGDC

A tight link between α-KGDC and OXPHOS is further exemplified when considering the role of pH in α-KGDC regulation. It has been widely shown that cytosolic Ca^2+^ elevation leads to rapid mitochondrial acidification, boosting oxidative metabolism. A range of pH between 6.6 and 7.4 has been demonstrated to increase α-KGDC activity [22]. Nonetheless, cytosolic pH is ~7.6 whereas in the mitochondrial matrix it ranges between 7.5 and 8.2 [34, 35]. The α-KGDC activity would thus be promoted upon environment acidification. In agreement with this, α-KG concentration in acidotic rat kidneys significantly decreases due to an increase of α-KGDC activity [36], which raises the question of whether changes in pH affect mitochondrial function in cancers, since they mostly rely on aerobic glycolysis and undergo a prominent acidosis [2]. However, cancer cells secrete lactate/H^+^ to maintain intracellular pH at physiological values, leading to the acidification of the extracellular microenvironment [37]. Noticeably, pH in the mitochondria is inherently related to the activity of the ETC that is driven by the proton motive force and Δψ_m_ [18]. Thus, the pH of the mitochondrial matrix would rather reflect the equilibrium between proton extrusion and entry into the matrix, mainly driven by OXPHOS activity [38]. It may be easily argued that a low OXPHOS activity is associated with a decrease of pH in the mitochondrial matrix due to proton accumulation, at least around the inner membrane. In this respect, the subsequent induction of α-KGDC activity and of the subsequent NADH generation might help maintaining a proper chemical gradient by fostering CI proton pumping in the IMS. Hence, beside the adenine nucleotide phosphorylation state, the NADH/NAD^+^ ratio and calcium levels, the OXPHOS might exert an additional control on the α-KGDC through the modulation of pH in the matrix of mitochondria.

#### ROS and α-KGDC activity

ROS are by-products of mitochondrial oxidative metabolism and their levels are a reliable indicator of ETC damage [39]. Noteworthy, α-KGDC can both sense and generate ROS (Fig. 1). An increase in ROS levels may decrease or completely inhibit α-KGDC function via two different mechanisms involving the modification of LA. While a post-translational modification may lead to the partial and reversible inhibition of the enzyme, the generation of a thiyl radical on the cofactor precedes its complete inactivation [40–42]. On the other hand, in response to NADH accumulation, stimulated by increased α-KG levels, the E3 subunit may generate H_2_O_2_ [43, 44] (Fig. 1) at much higher levels than CI [45]. Although physiological amounts of ROS are essential for cell survival, their excess fosters cancer initiation and progression through the induction of genomic instability, gene expression modifications, and the activation of signaling pathways [46, 47]. The aconitase is the most ROS-sensitive TCA cycle enzyme [48], and its inhibition in cells may therefore limit NADH production by interrupting the cycle from pyruvate to α-KG, and thus electron flux through the respiratory chain, ultimately reducing ROS in a negative loop. Conversely, cancer cells’ high reliance on glutamine metabolism may allow the α-KGDC to fully sustain NADH-linked respiration [49], even upon a great reduction of aconitase activity. However, unlike the latter, high ROS levels are required to inhibit the α-KGDC [42]. In this light, the elevated threshold might play a prominent role in the progression of tumors with ETC defects. Similarly, prolonged metabolic perturbations such as NADH and α-KG accumulation may lead to α-KGDC-dependent oxidative stress, which in turn may profoundly affect cancer cell redox state and metabolism through a ROS-mediated self-inactivation of the enzyme [50]. Based on these characteristics, with the aim of proposing potential anti-cancer treatment, Stuart and co-workers described a member of anti-cancer lipoate derivatives, CPI-613, which induces an E3-mediated burst of ROS leading to E2 inactivation in cancer cells [51]. Although CPI-613 is known to be implicated in cell death, whether this is due to α-KGDC inhibition, ROS overproduction, or to the reduction of the activity of other lipoate-dependent mitochondrial metabolic enzymes remains unclear [51, 52].

### The α-KGDC in cancer metabolic reprogramming

**Fig. 2:**
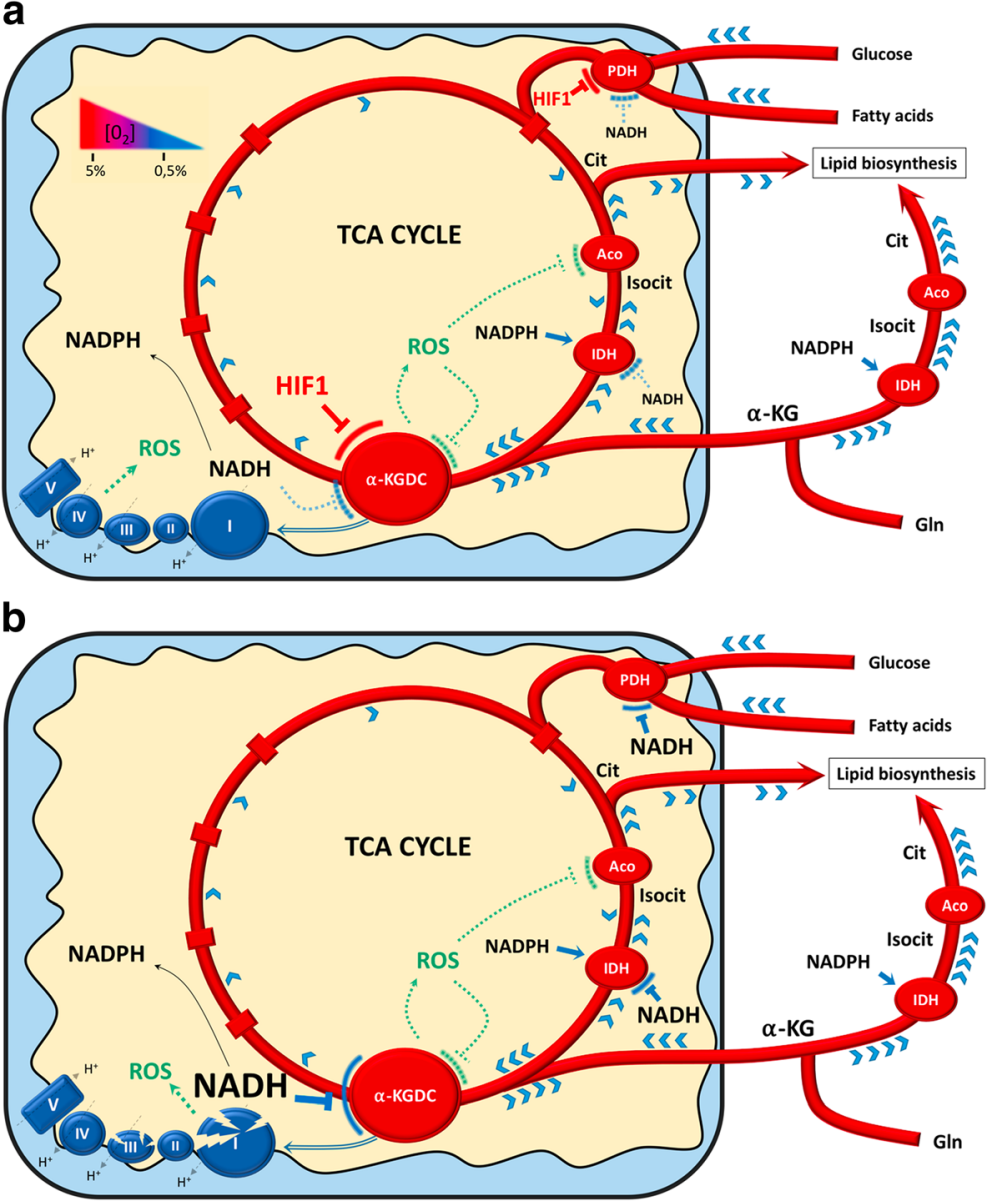
Scheme of the molecular actors driving α-KG reductive carboxylation in mitochondria upon hypoxia (**a)** and in cancer cells with ETC defects (**b**). Reductive carboxylation requires the elevation of the α-KG/citrate ratio and reduces α-KG to isocitrate that is subsequently converted to citrate. This latter is shuttled to the cytosol where it is used for the biosynthesis of lipids. Noteworthy, in both cases (**a-b**), ROS production by ETC and α-KGDC may induce the inhibition of aconitase and α-KGDC, thereby preventing citrate and α-KG oxidation, respectively. **a** Upon hypoxia (5–0.5% O_2_ tension), HIF1 can participate to the increase of the α-KG/citrate ratio, by preventing both PDH and α-KGDC activity, in turn limiting citrate production and α-KG oxidation, respectively. (﻿**b**) In cancer cells with ETC defects, the accumulation of NADH may lead to the inhibition of mitochondrial NADH-dehydrogenases (PDH, IDH, and α-KGDC), thus decreasing citrate production/oxidation and α-KG oxidation. Further, NADH increase may also promote NADPH-dependent IDH1/2 activity. Finally, it is important to note that NADH accumulation might also foster reductive carboxylation under low oxygen tension. The TCA metabolic flux is represented by *blue arrows* (), and () indicate the specific enzyme for each TCA cycle step. *Ac-CoA (Acetyl-Coenzyme A*; Aco (aconitase); Cit (citrate); Isocit (Isocitrate); Gln (Glutamine); α-KG (α-ketoglutarate)

Glucose and glutamine are the two primary nutrients utilized by cancer cells [11]. However, unlike glucose, which only provides carbon for biosynthesis, glutamine can provide both carbon and nitrogen for anabolic reactions, thereby conferring substantial additional benefits [53]. Cancer cells also depend on lipid biosynthesis for biomass increase [54–56]. A crucial precursor of fatty acids biosynthesis is the citrate that is canonically provided by glucose and glutamine metabolism through a forward running TCA cycle [10]. Instead, in neoplastic cells under hypoxic condition or in the presence of ETC defects, citrate is generated from reductive carboxylation of glutamine-derived α-KG, by cytosolic and mitochondrial NADPH-dependent IDH1 and 2, respectively [12, 13]. The α-KG conversion to isocitrate implies a lower α-KGDC activity and the unbalance of the α-KG/citrate ratio, leading to a TCA cycle functioning in a reverse mode, ultimately supporting de novo fatty acids synthesis and favoring tumor growth [13, 56, 57] (Fig. 2). It is worth noting that the activity of the α-KGDC is reduced via the hypoxia inducible factor 1 (HIF1)-mediated degradation of a splice variant of the E1 subunit, whose prevention renders cancer cells dependent on citrate or exogenous lipids to proliferate, and impedes tumor growth in vivo [56]. HIF1, the master regulator of the hypoxic response in malignant cancer cells [58], might also contribute to increase the α-KG/citrate ratio by inhibiting the activity of PDH and therefore citrate generation [59, 60] (Fig. 2a). These findings highlight an important role for HIF1 in modulating TCA metabolites levels and in the rewiring of α-KG fate from oxidative to reductive metabolism. In line with this, it has been shown that, in normoxia, constitutive activation of HIF1 alone is able to promote the reductive carboxylation of α-KG [12, 57]. Nevertheless, HIF1 is not essential in driving the reduction of α-KG since any conditions leading to a high α-KG to citrate ratio might promote it, by mass action on the TCA cycle flux [14]. For instance, in hypoxia and in cancer cells with ETC defects, the decrease of α-KG oxidation may be driven by changes in the levels of reducing equivalents. In this scenario, modifications of the mitochondrial redox state might account for the inhibition of the TCA dehydrogenases α-KGDC and PDH and for the promotion of the NADPH-dependent IDHs [12, 14, 61, 62] (Fig. 2b). Furthermore, ETC defects or hypoxia often lead to increase in ROS [63], which may contribute to the reduction of α-KGDC and aconitase activity. In this condition, the ROS-mediated inhibition of α-KGDC might foster α-KG accumulation and its diversion towards lipid biosynthesis, while the inactivation of aconitase might contribute to the accumulation and the extrusion of citrate from mitochondria [64] (Fig. 2).

Silencing *OGDH* in cancer cells with mitochondrial defects has been shown to prevent the production of sufficient levels of NADH, ultimately increasing the NADP^+^/NADPH ratio and preventing the NADPH-dependent IDHs to reduce α-KG [13]. This observation demonstrates that a minimal activity of α-KGDC is essential for the occurrence of reductive carboxylation and that this enzyme may operate even in the presence of respiration defects. Accordingly, HIF1 does not mediate a complete inhibition of α-KGDC activity, but only up to approximately 60% [56]. Moreover, accumulation of α-KG represses HIF1α stabilization and its downstream pathway by fostering the activity of the metabolic sensor prolyl hydroxylases (PHDs). This may represent a feedback control to keep the enzyme under the stringent regulation required for metabolic adaptation to hypoxia. To the same extent, the NADH and ROS-mediated regulation of α-KGDC activity represents additional feedback mechanisms that prevent complete enzyme inactivation. Since the forward and reverse modes of the TCA cycle are not exclusive, a fine-tuning of α-KGDC activity is required to balance α-KG fate in both energetic and anabolic pathways, according to oxygen levels and to ETC status.

In great contrast with impaired respiration, conditions of nutrient deprivation lead to OXPHOS enhancement and increase the NAD^+^/NADH ratio in the matrix, hence promoting mitochondrial biogenesis, fatty acids oxidation, and preventing oxidative stress [65, 66]. Surprisingly, a decrease in α-KGDC activity and thereby of mitochondrial ATP synthesis, due to the accumulation of α-KG and subsequent inhibition of the ATP synthase, has been shown to mimic calorie restriction in *Caenorhabditis elegans* [67]. This mechanism is proposed to ensure energetic efficiency in response to nutrient deprivation [67] and suggests that decreasing α-KGDC activity is not in contradiction with optimal cellular respiration. However, the accumulation of α-KG in starved animals appears to come from an increase in glutamine metabolism to sustain anaplerotic gluconeogenesis from amino acids catabolism and not from a decrease in α-KGDC activity [68]. Thus, the relevance of this mechanism has yet to be proven in cancer cells, where nutrient requirements and metabolic networks are known to be drastically different from non-malignant cells. In cancer cells, glutaminolysis exceeds the cellular requirement for glutamine in the production of amino acids, nucleotides, and energy [69]. Duràn and co-workers have shown that α-KG levels are a crucial sign of amino acids availability status. In this scenario, high cytosolic levels of α-KG may promote mammalian target of rapamycin 1 (mTORC1) signaling, which in turn blocks autophagy, the housekeeping mechanism to survive nutrient deprivation stress, and increases anabolism in neoplastic cells. On the other hand, low levels of α-KG have opposite effects and correlate with reduced mitochondrial respiration and ATP levels [70, 71]. In this light, it might be hypothesized that the high α-KG production due to enhanced glutamine metabolism might be beneficial for cancer cells by promoting proliferation while inhibiting autophagy [72]. However, the complex interplay between glutamine metabolism and the regulation of mTOR and autophagic processes in cancer cells makes an uncertainty whether α-KG plays a pivotal role in this respect [73]. In addition, upon glucose deprivation, treatment with α-KG derivatives and its reduced form 2-hydroxyglutarate (2-HG) has revealed the ability to inhibit the ATP synthase, resulting in mTOR signaling reduction and autophagy blockage in cancer cells [74]. Overall, these findings suggest that α-KG levels variation may differently affect autophagy regulation according to nutrient availability and compartmentalization of the metabolite (i.e., cytosolic versus mitochondrial), whose regulation still warrants investigation.

### Is α-KG an oncometabolite?

Mutations in fumarate hydratase (*FH*), succinate dehydrogenase﻿ ﻿(*SDH*)﻿﻿, and *IDH1* and *IDH2* are associated to specific human neoplasms that hence accumulate succinate, fumarate, and (*R*)-2-HG, respectively, all conveying broad oncogenic signals [75]. Mutations in *FH* and *SDH* follow the classic Knudson “two-hit” model, with somatic loss of gene function leading to the accumulation of their substrates. In a non-canonic fashion, a single allele mutation in *IDH1/2* creates a neomorphic enzyme with increased affinity for α-KG, from which an excess of the (*R*)-2-HG metabolite is produced [75, 76]. The prime mechanism of action of these so-called “oncometabolites” lies within the fact that they are structurally and metabolically similar to α-KG and retain the capacity to regulate a family of more than 60 enzymes involved in fatty acid metabolism, collagen biosynthesis, nutrient sensing, oxygen sensing, and epigenome editing [77, 78]. These enzymes are the Fe(II)/α-KG-dependent dioxygenases, and they include the PHDs introduced earlier. They are ubiquitously expressed and catalyze hydroxylation reactions on several targets. Moreover, they all use α-KG and O_2_ as co-substrates and require Fe(II) as a cofactor to produce succinate and CO_2_ [79]. Additionally, ascorbate is required to induce the reduction of Fe(III) to Fe(II), thus restoring enzyme activity [80, 81]. Noticeably, even in the absence of O_2,_ α-KG alone is sufficient to promote the activity of a subset of Fe(II)/α-KG-dependent dioxygenases, [82], which are instead inhibited by succinate and fumarate [83]. (*R*)-2-HG occupies the same binding site of α-KG and thereby acts as a competitive inhibitor [84]. Since the K_M_ of dioxygenases for α-KG is close to the metabolite physiological concentration, any condition causing even a modest variation in the cytosolic levels of α-KG may profoundly modify dioxygenases-mediated signals.

#### Understanding α-KG dynamics and their effect on its downstream targets

**Fig. 3:**
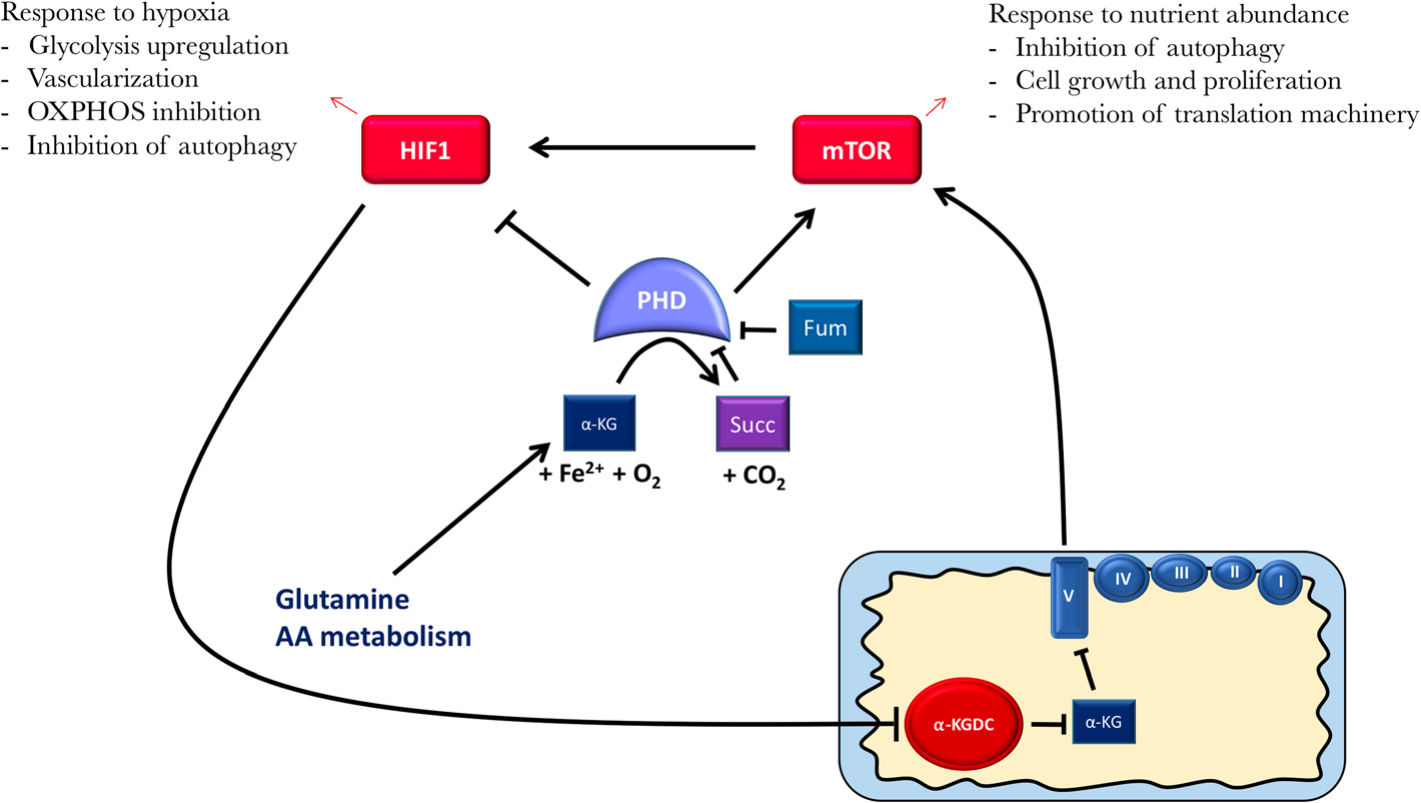
Schematic representation of the influence of α-KG levels and compartmentalization on PHDs and ATP synthase. PHDs have a dual role in the context of cell response to resources availability. PHDs control both adaptations to reduced oxygen levels, through HIF1α destabilization, and to nutrient availability via mTOR promotion. Cytosolic accumulation of α-KG may promote both PHDs and mTOR pathway and may inhibit HIF1 signaling. Conversely, mitochondrial accumulation of α-KG may prevent mTOR activation through the ATP synthase inhibition

**Fig. 4:**
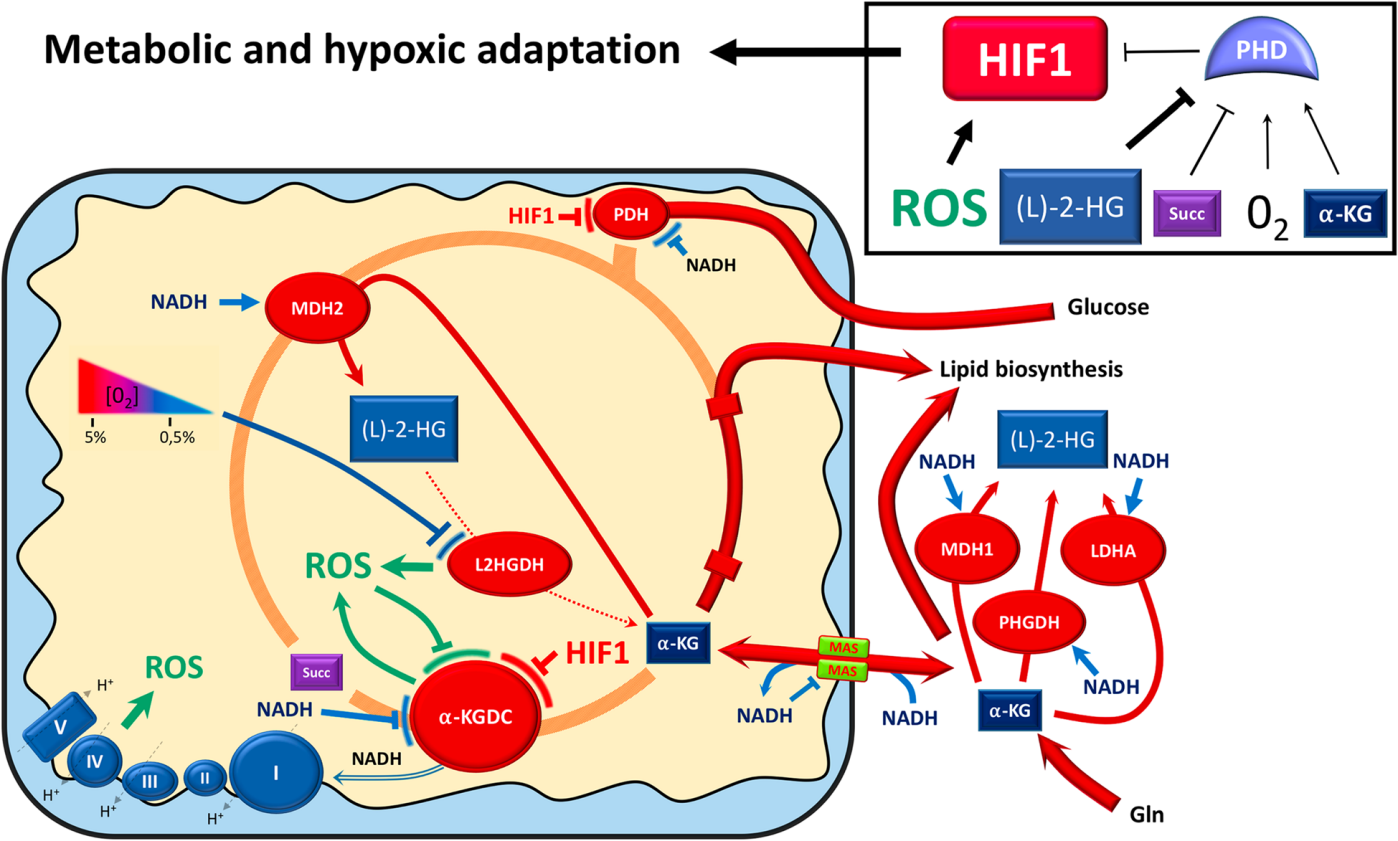
Mitochondrial signals that shape the HIF1-mediated metabolic and hypoxic adaptation in cancer cells. The hypoxic response may originate both from HIF1α stabilization, via the decrease in oxygen levels and the increase in the (*L*)-2-HG/α-KG ratio that lower PHDs activity, and from HIF1 activation, due to high ROS levels. In the first instance, reduced oxygen levels may inhibit the L2HGDH activity thereby decreasing (*L*)-2-HG conversion into α-KG, together with fostering ROS production. Additionally, the elevation of NADH levels in the mitochondria that stems from decreased respiration inhibits the malate-aspartate shuttle (﻿MAS) and therefore mitochondrial NADH production from cytosolic reductive power. Both mitochondrial and cytosolic NADH accumulation foster the activity of the NADH-dependent enzymes MDH1/2, PHGDH and LDHA that convert α-KG into (*L*)-2-HG. Furthermore, the potential production of ROS by the ETC and α-KGDC might further promote HIF1 activation. Finally, both NADH and HIF1 may inhibit α-KGDC and PDH, further promoting α-KG accumulation, in turn converted into (*L*)-2-HG or utilized for lipid biosynthesis

A comprehensive identification of enzymes that use α-KG to carry out their reactions will help to better define the metabolite role in bridging mitochondrial function to metabolic reprogramming in cancer cells. In this frame, PHDs have been extensively studied, in particular for their role in the response to hypoxia by determining HIF1α stabilization (Fig. 3). The transcription factor HIF1 consists of an α- and a β-subunit, which are both constitutively produced. Under normoxic conditions, HIF1α is degraded following the hydroxylation of two specific prolyl residues in its oxygen-dependent degradation domain (ODDD), operated by three different enzymes, namely PDH1, 2, and 3. This in turn recruits an E3 ubiquitin ligase complex containing the Von Hippel Lindau protein (pVHL), resulting in HIF1α ubiquitylation and subsequent degradation by the proteasome. Conversely, in hypoxia, the PHDs low affinity for oxygen does not allow them to hydroxylate HIF1α, which cannot be degraded and becomes stabilized. Although the three PHDs have been reported to be competitively inhibited by several TCA metabolites and pyruvate [83, 85–88], the most consistent effects are observed with fumarate and succinate [88] (Fig. 3). Accordingly, chronic inhibition of PHDs in both SDH-deficient and FH-deficient tumors is associated with the stabilization of HIF1α and activation of downstream hypoxic pathways even in normoxia (i.e., pseudohypoxia) [87]. Nevertheless, it is yet unclear whether the pseudohypoxic response caused by TCA cycle defects is sufficient *per se* to promote tumorigenesis or to support tumor progression [75]. Accumulation of α-KG, on the other hand, may have an opposite effect and rather lead to the constitutive destabilization of HIF1α. Accordingly, Gottlieb and his group have shown that the increase in α-KG alone is sufficient to oppose succinate, fumarate, and hypoxia-mediated activation of HIF1α. This resulted in the reversal of enhanced glycolysis and cell death [82, 89]. Hence, the levels of α-KG, even in hypoxia, may be sufficient to foster PHD activity and prevent the hypoxic response. In line with this, our group has demonstrated that cancer cells with severe mitochondrial CI impairment display an increase of α-KG/succinate ratio, likely due to an inhibition of α-KGDC resulting from an increased NADH/NAD^+^ ratio. This accumulation is associated to a constitutive destabilization of HIF1α even in a hypoxic environment (i.e., pseudonormoxia), together with a reduction of the tumorigenic potential in vivo [62, 90–92]. Importantly, both mitochondrial impairment and hypoxia can lead to the overproduction of L-(*R*)-2-HG, which is mostly generated by the conversion of glutamine-derived α-KG and competes with the latter [12, 61, 93–95]. Thus, dioxygenases would respond to the (*L*)-2-HG/α-KG ratio rather than to α-KG levels. Unlike (*R*)-2-HG, the L enantiomer does not stem from *IDH1* or *IDH2* mutations, but via a promiscuous activity of lactate dehydrogenase A (LDHA), MDH1 and PHGDH in the cytosol, and via MDH2 activity in the mitochondria, in a NADH-dependent manner [93, 96–98]. Hence, changes in the redox state as NADH accumulation and the subsequent modification of enzymatic functions associated to impaired respiration would foster α-KG conversion into (*L*)-2-HG. It has recently been remarked that an essential role of the respiratory chain is to transfer electrons from reduced substrate to oxygen, thereby maintaining an adequate redox state to allow aspartate synthesis and sustain cancer proliferation [26, 99]. In this context, it is plausible to envision that the α-KG may alternatively accept electrons and become reduced to (*L*)-2-HG, behaving as a *de facto* substitute of the ETC to support cancer growth. Consistently with this, supplementation with α-KG or inhibition of α-KGDC in normoxia causes a slight increase in (*L*)-2-HG levels, whereas in hypoxia the increase is far higher [93]. In the presence of respiration defects, on the other hand, the subsequent accumulation of α-KG might have different effects depending on its conversion rate into (*L*)-2-HG, as the enantiomer may inactivate PHDs over a certain threshold. It appears unlikely that the reduction of OXPHOS alone is sufficient to drive the (*L*)-2-HG-mediated HIF1 response, since, as shown by our group, a severe CI impairment rather prevents HIF1α stabilization. Additional factors would thus be required (Fig. 4). Besides low PHD activity due to the oxygen shortage, additional mechanisms may be envisioned. For instance, in physiological situations, the (*L*)-2-HG dehydrogenase (L2HGDH) converts (*L*)-2-HG back to α-KG [100], whereas in hypoxia *L2HGDH* expression is decreased by 50% and is therefore important to maintain a high (*L*)-2-HG/α-KG ratio (Fig. 4). Furthermore, a low L2HGDH activity would promote the production of ROS [93] with consequent HIF1α stabilization [101] (Fig. 4). It is remarkable that the regulation of *L2HGDH* expression is HIF1-independent, thus suggesting a potential upstream role in the initiation of the hypoxic response. Strikingly however, a recent study carried out by Burr and co-workers has shown that disruption of OGDH leads to HIF1α accumulation in normoxia via a ROS-independent PHD2-mediated mechanism that relies on (*L*)-2-HG production through both MDH1/2 and LDHA [102]. Hence, while cell response to hypoxia is mediated by multiple signals, including O_2_, reductive equivalents, α-KG, (*L*)-2-HG and ROS, the modulation of α-KGDC activity alone may have a great impact on HIF1α stabilization. To support this, it was shown that decreasing PHD activity does not inevitably promote the HIF1-signaling pathway, even in hypoxia. This seems to depend on a diminished HIF1α translation, due to a decreased mTOR signaling that follows O2 and amino acids deprivation, and hence, low levels of cytosolic α-KG, as PHDs are enhancers of mTOR activity (Fig. 3) [71]. Both low and high levels of α-KG may therefore impede the hypoxic response, suggesting that the maintenance of a certain α-KG amount is essential for HIF1 activation. In this light, it is possible to speculate that maintaining α-KGDC activity in an appropriate range would not only permit cells to meet metabolic requirements in hypoxia but it would also accurately control α-KG and (*L*)-2-HG levels in order to provide cells with a tailored signal for HIF1 activation.

Besides mTOR, autophagy is also modulated by HIF1 [103], making PHDs and thereby α-KG levels pivotal in organelles catabolism. Accumulation of α-KG may also promote mTOR function via ATP synthase inhibition in the mitochondrion, without involving PHDs [67] (Fig. 3). Hence, while cytosolic accumulation of α-KG might prevent autophagy by activating PHDs, the elevation of its levels in the mitochondria would instead promote it, via ATP synthase inhibition. Nonetheless, cytosolic Fe(II)/α-KG-dependent dioxygenases respond to the ratio between α-KG and its various competitors, whereas α-KG mediates the inhibition of ATP synthase in a non-competitive manner [67]. Consequently, the former mechanism is sensitive to high cytosolic α-KG/2-HG ratio, generated by an increase in amino acids metabolism or a decrease in OXPHOS. The latter may instead be triggered by both α-KG and its reduced forms in the mitochondrial matrix with respect to ATP synthase abundance, and would serve to optimize cell respiration according to substrate availability, thereby contributing to caloric restriction adaptation.

In conclusion, the response to fluctuations of α-KG levels in cells is multifactorial and remains an open area of research. The α-KG signaling is likely to be defined by the metabolite abundance, its oxidation state, and dynamics, which are determined by ETC status and oxygen levels, perhaps among other yet unknown mechanisms. Based on these considerations, it is plausible to argue that fluctuations of α-KG levels may be an intrinsic characteristic of tumor progression, useful to trigger the bioenergetic changes in response to selective pressures. In this light, the role of α-KG remains dual as it may promote both oncogenic and tumor suppressive functions, paralleling the oncojanus function of mitochondrial genes, as we have previously proposed [91].

### Impact of α-ketoglutarate on cancer cell epigenetics

Epigenetics alterations at both DNA and histone levels are increasingly being recognized as modifiers of tumorigenesis [104]. CpG islands are widely hypermethylated in many cancer types compared to the corresponding normal tissue, while the rest of the genome is rather subject to demethylation. The hypermethylation of CpG islands has been utilized as a criterion to distinguish different tumor types from non-malignant tissue [105], and tumors characterized by high levels of DNA methylation have been classified as having a CpG island methylator phenotype and are predominantly associated with worse prognosis, potentially due to a silencing of tumor suppressor genes. In many cases, this phenotype originates in early phases of tumorigenesis of many tumor types such as glioblastomas, acute myeloid leukemias, gastric cancer, and ependymomas [106–111], where drugs targeting the DNA methylation machinery are a promising strategy.

The large family of α-KG-dependent dioxygenases includes two classes of enzymes involved in demethylation and hydroxylation reactions of DNA and histones. The ten-eleven translocation hydroxylases (TET 1 to 3) catalyze DNA demethylation, whereas the Jumonji C domain containing lysine demethylases (KDM 2 to 7) is the largest family of histone demethylases [112–114]. Both (*L*)-2-HG and (*R*)-2-HG are competitive inhibitors of TETs and KDMs, and are thus important modifiers of the epigenetic landscape of cancer cells [84, 94, 115, 116]. Accordingly, accumulation of (*L*)-2-HG and (*R*)-2-HG has been associated to several types of cancers [96, 117–119]. Similarly, recent studies have revealed that together with 2HGs, succinate and fumarate can also induce alterations in DNA and histones methylation, thus enhancing cancer formation [84, 120–125]. These findings suggest that different cytosolic concentrations of α-KG affect the methylation status of both histones and DNA and thereby trigger epigenetic changes. Accordingly, Thompson’s group has demonstrated how maintenance of a proper α-KG to succinate ratio is fundamental to determine the identity and the fate of embryonic stem cells (ESC) [126]. In particular, a high α-KG/succinate ratio promotes the activity of DNA and histone demethylases, and modifying this ratio is sufficient to regulate multiple chromatin modifications. Indeed, treatment with α-KG supports ESC self-renewal, which is known to display an unusual “open” chromatin structure, associated to hypertranscription [127]. In this light, high cytosolic levels of α-KG would promote high energy-consuming processes, a hypothesis that is supported by the existence of the PHD-driven mTOR activation mediated by α-KG, which fosters anabolic processes. Conversely, in cancer cells facing hypoxia, it is plausible that α-KG conversion into (*L*)-2-HG most likely helps in reducing the energetic demand while promoting HIF1α stabilization for hypoxic adaptation. Consistent with this, hypoxia induces a global increase in trimethylation of histone H3 at lysine 9 (H3K9me3) marks, known to repress gene expression, through the accumulation of (*L*)-2-HG that inhibits the activity of the demethylase KDM4C [94]. Furthermore, oxygen shortage has recently been shown to directly cause DNA hypermethylation by reducing TET activity in cancer cells, predominantly at the level of gene promoters [128]. Notwithstanding this, both TETs and KDMs may stimulate the transcription of specific HIF1-targeted genes, while being themselves transcriptional targets of HIF1 [125, 128–133], a mechanism that most likely compensates for their lower enzymatic activity. Hence, while oncometabolites and low oxygen availability can promote a closed chromatin state and a drop in global gene expression through α-KG-dependent dioxygenases activity, it is plausible that retaining a minimal activity of these enzymes would induce a specific genetic response in cells by restraining transcription machinery to HIF1-targeted genes.

Similarly, given the role of α-KG as an indicator of amino acids availability, it is plausible to speculate the occurrence of an epigenetic remodeling upon glutamine deprivation, which may be faced by solid cancers. Accordingly, a recent study has demonstrated that glutamine deficiency is associated to low α-KG levels, which may in turn determine the inhibition of KDMs in the core regions of the tumor. In this context, the increase in histone methylation induces cancer cells dedifferentiation and may cause therapy resistance [134].

The consequence of epigenetics modifications is the transduction of external stimuli into a transcriptional response, thus adjusting cells phenotype without affecting their genotype [135]. It is most likely that cell bioenergetic changes driven by external and internal selective pressures promote an intricate epigenetic remodeling through α-KG signaling.

## Conclusions

A revisited role of mitochondria highlights that they are not mere bystanders during carcinogenesis. The ever-changing tumor microenvironment may force cells to rely on fluctuating levels of oxygen, as well as varying availability and types of nutrients, whereby optimization of substrates utilization and a continuous restructuring of both metabolic and genetic signatures becomes mandatory for survival. In this review, we have highlighted a hub role for the TCA cycle enzyme α-KGDC as a front-line player in the adaptation of cancer cells to a demanding environment in vivo. This enzyme may be considered a gatekeeper of the OXPHOS system and one of the major regulators of mitochondrial metabolism. Indeed, α-KGDC responds to OXPHOS activity fluctuations, controls the mitochondrial redox status through NADH and ROS levels balance, and directs the TCA metabolite fluxes towards energetic, anabolic, and signaling pathways. Changes in α-KGDC activity, and consequently in overall mitochondrial bioenergetics, may impact not only on TCA cycle fluxes but may become amplified and eventually drive an intricate metabolic and epigenetic remodeling. Overall, NADH/NAD^+^ and AMP/ATP ratio, oxidative stress, membrane potential, and oxygen levels are pivotal players in the translation of the α-KG signal. In turn, α-KG and its reduced forms may influence the activity of dioxygenases to shape cells metabolic and epigenetic landscape according to oxygen and nutrient availability and ETC efficiency. In this light, the α-KGDC and its substrate appear to be inescapable actors in cancer cells plasticity. It is remarkable that genetic and metabolic modifications within a tumor mass are likely to differ from cell to cell thereby contributing to a phenotypic heterogeneity, thus accounting for therapy resistance and disease progression [134, 136]. Several studies have considered the anti-tumorigenic properties of α-KG but its mechanisms of action are still not fully understood. The anti-tumorigenic effect observed upon in vivo treatment with derivatives of α-KG has been shown not only to depend on impaired HIF1 signaling pathway but also to a much greater extent on multiple unknown side effects of α-KG on tumor growth [94]. In this frame, it has been reported that α-KG antagonizes the effect of other oncometabolites, and might therefore be considered a tumor suppressor metabolite. Nevertheless, α-KG supplementation leads to the risk of feeding oncogenic pathways not only due to its conversion into (*L*)-2-HG in respiratory deficient cells but also into succinate and fumarate, on a long-term treatment [137]. On the other hand, several human Fe(II)/α-KG-dependent dioxygenases have been investigated as possible therapeutic targets and might represent an interesting alternative strategy to render tumors more sensitive to radiotherapy and chemotherapy [90]. A complete understanding of the α-KG-mediated interplay between metabolic and genetic reprogramming will help us to disclose a new therapeutic window in which cancer progression can be restrained. While cancer is defined as a genetic disease, there is nowadays a growing and legitimate interest in its metabolic dimension. In particular, these two aspects are increasingly recognized as being so interconnected that they are likely to represent the two edges of the same sword.

## Abbreviations

2-HG: 2-Hydroxyglutarate
Ca^2+^: Calcium
CI: Complex I
CoA: Coenzyme A
CS: Citrate synthase
DHLA: Dihydrolipoic acid
E1: α-﻿Ketoglutarate dehydrogenase
E2: Dihydrolipoamide succinyltransferase
E3: Dihydrolipoamide dehydrogenase
ETC: Electron transport chain
FH: Fumarate hydratase
H3K9me3: Trimethylation of histone H3 at lysine 9
HIF1: Hypoxia inducible factor 1
IMM: Inner mitochondrial membrane
IMS: Mitochondrial intermembrane space
KDM: Lysine demethylases
K_M_: Michaelis constant
L2HGDH: (*L*)-2-HG dehydrogenase
LA: Lipoic acid
MAS: Malate-aspartate shuttle
mtDNA: Mitochondrial DNA
mTORC1: Mammalian target of rapamycin 1
ODDD: Oxygen-dependent degradation domain
OGC: 2-Oxoglutarate carrier
OXPHOS: Oxidative phosphorylation
PDH: Pyruvate dehydrogenase
PHDs: Prolyl hydroxylases
Pi: Inorganic phosphate
pVHL: Von Hippel Lindau protein
ROS: Reactive oxygen species
Succ-CoA: Succinyl coenzyme A
TCA: Tricarboxylic acid
TET: Ten-eleven translocation hydroxylases
TOR: Target of rapamycin
TPP: Thiamine pyrophosphate
α-KG: α-Ketoglutarate
α-KGDC: α-Ketoglutarate dehydrogenase complex
Δψ_m_: Membrane potential
